# Accounting for the effect of GERD symptoms on patients’ health-related quality of life: supporting optimal disease management by primary care physicians

**DOI:** 10.1111/j.1742-1241.2007.01586.x

**Published:** 2007-12

**Authors:** N W Flook, I Wiklund

**Affiliations:** 1 University of Alberta Family Medicine Clinic Edmonton, AB, Canada; 2 AstraZeneca R&D Mölndal, Sweden

## Abstract

**Aim:**

To review, from a primary care physician (PCP) perspective, the use of patient-reported outcome (PRO) instruments for assessment of gastro-oesophageal reflux disease (GERD) symptoms, their impact on health-related quality of life (HRQL) and the effectiveness of therapy.

**Results:**

While generic and disease-specific PRO instruments have been used in the assessment of GERD, the latter can be considered to be more appropriate as they focus only on problems relevant to the disease in question (and therefore tend to be more responsive to change). Such instruments include the Quality of Life in Reflux and Dyspepsia (QOLRAD) questionnaire and the Gastrointestinal Symptom Rating Scale and the Reflux Disease Questionnaire (RDQ). Their use indicates that GERD symptoms are troublesome and significantly reduce patients’ HRQL, and that effective treatment of GERD improves HRQL. The GERD Impact Scale (GIS) questionnaire, primarily developed for use within primary care, can also help to determine the impact of symptoms on patients’ everyday lives and, in turn, the benefit of appropriately targeted therapy. Notably, these PRO instruments were developed from focus groups of GERD patients, and only aspects rated of highest importance are used in the final instruments. Consequently, PCPs can feel confident that these questionnaires encompass the most relevant points that they are likely to ask in terms of how symptoms affect patients’ everyday lives.

**Conclusions:**

Primary care physicians are encouraged to make wider use of PRO instruments within routine practice to improve communication with their GERD patients that, in turn, could lead to improved clinical outcomes and greater patient satisfaction.

**Review Criteria**

- We conducted a review of validated patient-reported outcome (PRO) instruments used in gastro-oesophageal reflux disease (GERD), based on the authors’ expertise in the field and a supplementary MEDLINE search with the terms ‘health-related quality of life’, ‘quality of life questionnaire’, ‘patient-reported outcomes’, ‘patient satisfaction’ and ‘gastro-oesophageal reflux disease’.
- We reviewed papers reporting health-related quality of life (HRQL) data in patients with GERD, including the effect of GERD on HRQL, treatment efficacy and HRQL, patient satisfaction and physician-patient agreement, to help primary care physicians to incorporate PRO instruments into their day-to-day management of patients with GERD.

**Message for the Clinic**

- An evidence-based review shows that PRO instruments can accurately assess the nature of GERD symptoms, their impact on HRQL and the efficacy of treatment.
- Primary care physicians are encouraged to make wider use of PRO instruments as part of their management of patients with GERD, given that such questionnaires can facilitate patient communication and help physicians understand and satisfy the therapeutic needs of their patients.
- Among validated PRO instruments, the GERD Impact Scale represents a practical tool that is easy for primary care physicians to incorporate into their everyday practice.

## Introduction

Gastro-oesophageal reflux disease (GERD) is one of the most common gastrointestinal diseases that primary care physicians (PCPs) encounter in daily practice, with up to 20% of the population of developed countries being affected by at least weekly reflux symptoms (1,2). This chronic and potentially serious condition results from continued exposure of the oesophageal mucosa to refluxed gastric contents, which carries a risk of erosive oesophageal tissue damage and subsequent (albeit relatively rare) complications, such as stricture and Barrett's oesophagus (3). However, many patients with typical GERD symptoms do not have endoscopically visible erosive disease (3–5), indicating that endoscopy (while highly specific for diagnosis of GERD) is of limited value in guiding disease management (6,7). Indeed, current guidelines recommend a symptom-based approach to the diagnosis of GERD in patients without ‘alarm’ features (i.e. those suggestive of cancer, such as persistent vomiting, bleeding, anaemia, dysphagia, weight loss and abdominal mass) (6–9).

Patients with GERD may present with a broad range of troublesome symptoms that can extend beyond the cardinal symptoms of heartburn and regurgitation (10). Symptoms can overlap with other gastrointestinal diseases (such as dyspepsia) (11), and may even include chest pain or extra-oesophageal manifestations, such as chronic cough and asthma (10,12). GERD symptoms are a major burden for many patients, in terms of disrupted physical, social and emotional well-being (13), leading an international expert panel to propose that GERD be defined as reflux symptoms sufficient to impair patients’ lives (8). Indeed, a recent global consensus on GERD has agreed upon a new definition that includes symptoms (and/or complications) that can be attributed to GERD and that are troublesome. This adds a patient-defined qualifier indicating that the severity of symptoms is sufficient to impair their quality of life and/or limit their function (10). In the present article, we review how patient-reported outcome (PRO) instruments can be used not only to assess this burden but also to support optimal management of GERD by PCPs.

## Patient-reported outcome instruments

**Figure 1:**
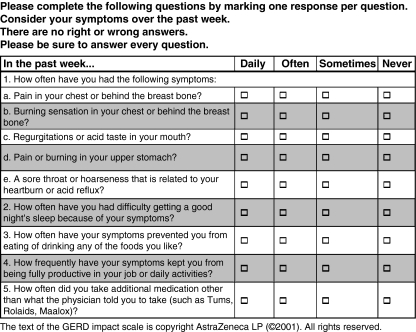
The GERD Impact Scale (available at: http://www.nexium.net/hcp/ScientificResources/The-GERD-Impact-Scale.aspx?mid=35)

Two basic types of PRO instrument can be used to measure health-related quality of life (HRQL): ‘generic’ and ‘disease-specific’. ‘Generic’ instruments are designed to evaluate functional status and well-being in general populations, whereas ‘disease-specific’ instruments focus only on problems relevant to the disease in question (14). Examples of the generic and disease-specific PRO instruments that have been used to assess GERD symptoms and their effect on HRQL are summarised in Table 1. Patients generally find these PRO instruments quick and easy to complete, which facilitates their use both in the research setting and for everyday primary care practice. Notably, however, disease-specific instruments are probably more appropriate for use in the day-to-day management of GERD patients, because they are not only specific to GERD (or gastrointestinal symptoms in general) but also tend to be more responsive to change. One example of a disease-specific PRO instrument is the fully validated Quality of Life in Reflux and Dyspepsia (QOLRAD) questionnaire (18), which was developed for patients suffering from upper gastrointestinal symptoms including heartburn and dyspepsia. QOLRAD monitors HRQL across a full range of clinically relevant aspects, such as emotional distress, sleep disturbance, and food and drink problems in the past week. Other disease-specific PRO instruments include the Gastrointestinal Symptom Rating Scale (GSRS) (19), which evaluates how patients perceive the severity of their gastrointestinal symptoms over the past week. The GSRS was originally developed in patients with irritable bowel syndrome and peptic ulcer disease, but has since been specifically evaluated for use in patients with GERD (22). The Reflux Disease Questionnaire (RDQ) (20), another self-administered questionnaire, was designed to assess the frequency and severity of heartburn, acid regurgitation and dyspeptic complaints (pain or burning in the upper stomach) over the past week. Such questionnaires have generally proved easy-to-use, although some take longer to complete than others and this could be a problem for some patients under certain circumstances. More recently, a one-page, patient-completed and short questionnaire known as the GERD Impact Scale (GIS) (21) (Figure 1) has been developed which is intended to quickly determine the burden and impact of GERD symptoms over the last week. Comprised of nine self-explanatory questions, for which patients tick one of four options to describe how often they are affected (‘daily’, ‘often’, ‘sometimes’ and ‘never’), the GIS can be completed by the patient without input from his/her physician, e.g. at the start of a consultation. Questions about symptom burden ask the patient to estimate the frequency of symptoms and the use of supplementary (over-the-counter) medication to control symptoms, while the impact of symptoms is assessed by inquiring about their effect on sleep and the ability to eat and drink. The adverse impact of GERD symptoms on the latter aspects of daily life, as well as work performance and concomitant high usage of over-the-counter medication, is not always recognised by clinicians and together indicate that GERD symptoms are prominent and far from being well-controlled. Hence the intent of using the GIS is not only to raise awareness of GERD symptoms and their impact but also to provide a simple tool to monitor how patients respond to treatment for their GERD symptoms. The GSRS, RDQ and the GIS therefore allow for the assessment of subjective HRQL in relation to physician-perceived symptom severity and frequency. This is particularly relevant considering that physicians often underestimate the severity and impact of their patients’ GERD symptoms (8). Notably, PRO instruments such as, QOLRAD, RDQ and GIS, were developed from focus groups of GERD patients, and only the items rated as being of the highest importance are used in the final instruments. Consequently, physicians can feel confident that these questionnaires encompass the most relevant points that they are likely to ask in terms of GERD symptoms and how they affect patients’ everyday lives.

**Table 1:** Patient-reported outcome instruments that have been used to assess the severity of gastro-oesophageal reflux disease (GERD) symptoms and the impact of symptoms on health-related quality of life

Instrument	Items and domains	Scoring system	Scoring interpretation
Generic			
SF-36 (15)	36 items grouped into eight domains(physical function, bodily pain, role limitations – physical, vitality, general health perceptions, social function, role limitations-emotional, mental health)	Scored from 0 (lowest well-being) to 100(highest well-being); two summary component scales (physical and mental)	Low score represents worst health state
PGWBI (16)	22 items grouped into six domains (anxiety,depressed mood, well-being, self control, general health, vitality)	Items scored on a 6-grade Likert-type scale(1, worst health to 6, best health). Overall worst possible score = 22; overall best possible score = 132	Low score represents worst health state
EQ-5D (17)	Five items concerning mobility, self-care,usual activities, pain/discomfort and anxiety/depression	Items scored on a 3-grade scale of worseninghealth (e.g. Mobility item: ‘no problems in walking about’ to ‘confined to bed’)	Higher grades represent poorer stateof health
Disease-specific			
QOLRAD (18)	25 items, five domains (emotional distress,sleep disturbance, food/drink problems, vitality, physical/social functioning)	Items scored on a 7-grade Likert-type scalewith regard to degree of distress (7, no to 1, a great deal of distress) and frequency of the problem (7, none of the time to 1, all of the time)	Low score represents more severe impacton daily functioning
GSRS (19)	15 items, five symptom clusters (reflux,diarrhoea, constipation, pain, indigestion)	Items scored on a 7-grade Likert-type scale(1, none at all to 7, very severe discomfort)	High score represents worse discomfort
RDQ (20)	12 items measuring the frequency andseverity of heartburn, regurgitation and dyspeptic symptoms	Items scored on a 6-grade Likert-type scale(did not have to daily for frequency; did not have to severe for severity)	High score represents more frequent andsevere symptoms
GERD Impact Scale (21)	Nine items measuring three distinct factors(burning and pain, other acid-related symptoms, impact of GERD symptoms)	Items scores on a 4-grade scale (never to daily)	Higher grades represent worse discomfort

EQ-5D, EuroQoL 5-dimensional health-related quality-of-life questionnaire; GSRS, Gastrointestinal Symptom Rating Scale; RDQ, Reflux Disease Questionnaire; SF-36, Short Form-36; PGWBI, Psychological General Well-Being Index; QOLRAD, Quality of Life in Reflux and Dyspepsia.

## Assessing the impact of GERD symptoms on HRQL

With the availability of validated questionnaires, the negative effect of GERD on HRQL is becoming better defined. Findings show that subjects with untreated GERD have lower HRQL than the general population (13,23–28). In the German ProGERD (Progression of Gastro-oesophageal Reflux Disease) study, for example, patients with symptoms of GERD had substantially impaired HRQL in terms of both physical and psychosocial aspects of well-being compared with the general German population. From a disease-specific point of view, patients felt restricted as a result of food and drink problems, disturbed sleep, and impaired vitality and emotional well-being (23). Similar findings were observed in other large-scale population surveys (13,24–28). A survey conducted by the National Heartburn Alliance in the USA, for example, found more than 70% of the respondents reported reduced enjoyment of food and that eating out was a problem because of their GERD symptoms (27). In addition, the majority reported that heartburn affected their sleep and caused problems with concentration at work, with over 30% stating that social activities were curbed by heartburn (27). Another large US general population survey found that respondents with nocturnal GERD symptoms reported significantly greater impairment of well-being than did respondents who reported having only daytime GERD symptoms (29).

Notably, impairment of HRQL strongly correlates with patient-perceived severity and frequency of GERD symptoms. In a random sample of the general Swedish population, for example, a decrease in well-being (as assessed using the Psychological General Well-Being Index) was significantly associated with the severity of heartburn (p < 0.0001), acid regurgitation (p < 0.05) and abdominal pain (p < 0.0001) based on the GSRS (30). Indeed, even symptoms that were rated as mild [i.e. GSRS score of ≤ 3 (the worse possible score being 7 on this scale)] had a clinically meaningful adverse effect on well-being. The Swedish study did not investigate the effect of symptom frequency on well-being. However, data reported elsewhere indicate that ≥ 2 days of mild symptoms per week are sufficient to impair HRQL in patients with GERD (8,31). These findings underscore the importance of incorporating an assessment of HRQL as part of the management of GERD in primary care, especially for newly diagnosed patients.

## Effective treatment improves HRQL in patients with GERD

**Figure 2:**
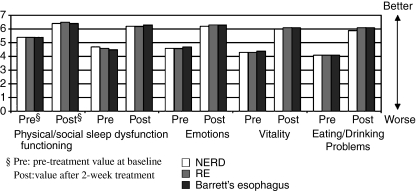
Quality of Life in Reflux and Dyspepsia (QOLRAD) questionnaire dimensions, assessed prior to treatment and after 2 weeks of treatment with esomeprazole, in German patients with symptoms suggestive of gastro-oesophageal reflux disease; p < 0.0001 for all changes vs. pretreatment (baseline) (23). Reprinted from Kulig M, et al. Quality of life in relation to symptoms in patients with gastro-oesophageal reflux disease – an analysis based on the ProGERD initiative. *Aliment Pharmacol Ther* 2003; 18: 767–76, with permission from Blackwell Publishing. RE, reflux oesophagitis (patients received esomeprazole 40 mg/day for 4 weeks in total); NERD, non-erosive reflux disease (patients received esomeprazole 20 mg/day for 2 weeks)

Effective medical management of GERD hinges on the physician being able to ensure that appropriately targeted treatment provides enduring relief from symptoms (and/or prevention of complications) and, in turn, improvement of HRQL. This objective can usually be accomplished by sufficient control of gastric acid secretion (32), and numerous studies show that successful treatment of symptoms with acid-suppressive therapy leads to marked improvement of HRQL (33–41). In this regard, the proton pump inhibitors (PPIs) are the most effective, first-line, initial and long-term therapy for the treatment of patients with GERD (6–9). The findings of the ProGERD study (23), for example, attest to the efficacy of PPI therapy for improving HRQL in patients with symptoms of GERD (Figure 2). After treatment, physical and mental aspects of well-being reached levels similar to those of the general German population, and the degree of symptom relief was one of the main factors associated with improvements in HRQL measures.

Once a patient with GERD is symptom-free, guidelines advocate that treatment can be stepped down either to the lowest effective PPI dose that controls symptoms or to less intensive acid suppression with a H_2_-receptor antagonist (6,7). HRQL was monitored as part of a study that investigated the use of the step-down approach in the long-term management of patients with symptoms of GERD (41). In this study, patients who were relieved of symptoms (≤ 1 day of mild GERD symptoms during the previous 7 days) after 4 weeks’ initial treatment with esomeprazole 40 mg/day were randomised to receive 6 months’ maintenance treatment with esomeprazole 20 mg/day, esomeprazole 20 mg on-demand or ranitidine 150 mg twice daily. HRQL, as assessed using the QOLRAD questionnaire, was impaired at baseline and improved significantly after initial treatment with esomeprazole. Thereafter, while ranitidine maintenance therapy was effective for maintaining HRQL in some patients, both esomeprazole maintenance regimens proved significantly more effective than continuous treatment with a H_2_-receptor antagonist for sustaining the improvement in HRQL seen during the symptom control phase (p < 0.0001 for all QOLRAD dimensions). Overall, daily esomeprazole therapy was more effective than esomeprazole on-demand in terms of enduring symptom relief (41). Ponce et al. (42) also noted the ability of on-demand therapy with a PPI to maintain HRQL improved with short-term healing therapy, but the findings of the latter study are somewhat limited by the absence of a comparative treatment arm of daily maintenance therapy.

Taken together, these findings outline how PRO instruments can be easily used by PCPs to monitor the response of GERD patients to appropriately targeted therapy.

## Improving patient-physician communication and addressing unmet patient needs

As noted above, it has become standard practice for PCPs to manage GERD empirically on the basis of symptom severity and frequency alone (6–9). Traditionally, symptom evaluation involves the physician asking appropriate questions to elicit information from the patient; the physician then interprets the response and makes a judgment as to the severity of symptoms. However, there is often poor agreement between patients and physicians in their assessment of GERD symptom severity, with physicians tending to underestimate symptom severity and overestimate treatment effects (43,44). In turn, physicians are likely to be failing to recognise the true HRQL impact of the patient's GERD symptoms. A separate study, for example, found that baseline QOLRAD scores were not strongly correlated with the physician assessment of overall disease severity (43). This failure to sufficiently account for symptom-related disability and the effect on HRQL, coupled with communication difficulties between physicians and patients (45), may therefore result in suboptimal disease management and contribute to treatment dissatisfaction. In the Patient Unmet Needs Survey (PUNS) of 11,064 chronic heartburn sufferers, for example, only 46.2% of respondents were totally satisfied with their current heartburn medication (46). Interestingly, respondents who reported complete heartburn resolution reported higher levels of total satisfaction (46). Other authors have found that the greater the improvement on the vitality domain of QOLRAD questionnaire during PPI therapy the more likely the patient was to be satisfied with the treatment (47), while those patients who stay on PPI therapy longer (presumably because of better symptom control) show higher levels of satisfaction with treatment (as determined by the GERD Treatment Satisfaction Questionnaire) (48). Therefore, the likes of the PUNS data and other findings linking HRQL outcomes and treatment satisfaction reinforce the need for more comprehensive assessments of symptoms severity and the effect on well-being, using PRO instruments, both at diagnosis and during pharmacotherapy to provide more GERD sufferers with enduring symptom relief.

Although a complete symptomatic response is quite feasible for most patients in the era of PPI therapy, it is not uncommon for PPI-treated GERD patients to have some residual symptoms. The extent to which patients experience persistent GERD symptoms during PPI therapy is not generally appreciated by physicians (49), who may tend to overestimate the success of this class of medications (43). The PASS (Proton pump inhibitor Acid Suppression) test is an example of a simple and clinically applicable tool that can help PCPs to identify undertreated patients and assess their response to a change in therapy. The PASS test is composed of five, clear, easy-to-understand (yes/no) questions relevant to patient experience (49). The utility of the PASS test was evaluated in patients with persistent GERD symptoms of at least mild severity despite PPI therapy, who went on to receive open-label treatment with esomeprazole 40 mg/day for 4 weeks (*n* = 249). Mean total PASS test scores, which were > 3 at the baseline evaluation (against a maximum possible score of 5), fell to ≤ 2.0 after 4 weeks’ esomeprazole therapy, and 30–33% of patients were PASS test responders (i.e. score of zero after esomeprazole treatment). PASS test responder effect sizes on the GSRS, RDQ and QOLRAD were, on average, two to three times higher than the effect sizes for PASS test non-responders. In other words, there is a high likelihood that if the patient has a PASS test score of zero (in this case, by switching to a different PPI with greater acid-suppressive efficacy) then their HRQL will have also been improved. The GIS has also proved of value in the identification of GERD patients with uncontrolled symptoms in need of more effective therapy (21). During validation of the questionnaire, for example, physicians reported altering their treatment decision in around one-third of patients (35%) based on information provided by the GIS (21).

Physicians should also consider that issues related to dosing and treatment adherence (compliance) may be involved when an incomplete response to PPI therapy is apparent and well-being continues to be impaired. Once the physician has confirmed that GERD symptoms are still present during PPI therapy, the challenge will be to determine whether treatment is being taken as prescribed. For example, the importance of treatment adherence and ingestion of PPI therapy before a meal should be stressed. If it can be confirmed that adherence is acceptable and that residual symptoms are in fact acid-related, e.g. with 24-h pH monitoring on medication to determine the presence of reflux (7), then the patient may benefit from an increase in acid-suppressive therapy. This may be sufficient to bring symptoms under control and, in turn, improve HRQL.

## Conclusions

As the role for PCPs in the management of GERD continues to evolve and expand, there is increasing potential for them to improve the value of their care services for GERD patients. A better understanding of each patient's personal experience of the disease, which can be easily captured with the use of PRO instruments, will help PCPs to appreciate that even mild symptoms of GERD can be troublesome and can be associated with a clinically relevant reduction in patient well-being. Indeed, HRQL is now a component of the definition of GERD and its improvement is considered to reflect successful therapeutic intervention. Consequently, a need exists for improved questioning during consultation and more effective and open communication to assist in eliciting the most relevant information from patients. This process can be augmented by the use of relevant PRO instruments such as the GIS, which is practical and easy to incorporate into everyday primary care practice. Primary care physicians are therefore encouraged to increase their knowledge of PRO instruments and make wider use of these valuable tools as part of their management of patients with GERD, given that such questionnaires can facilitate patient communication and help physicians understand and satisfy the needs of patients with GERD. In doing so, clinical outcomes may be improved and patient satisfaction increased, with strengthening of the relationships between patients and their physicians.

## References

[1] Dent J, El-Serag HB, Wallander MA (2005). Epidemiology of gastro-oesophageal reflux disease: a systematic review. Gut.

[2] Jones R (1995). Gastro-oesophageal reflux disease in general practice. Scand J Gastroenterol Suppl.

[3] McDougall NI, Johnston BT, Kee F (1996). Natural history of reflux oesophagitis: a 10 year follow up of its effect on patient symptomatology and quality of life. Gut.

[4] Armstrong D (1999). Endoscopic evaluation of gastro-esophageal reflux disease. Yale J Biol Med.

[5] Thomson AB, Barkun AN, Armstrong D (2003). The prevalence of clinically significant endoscopic findings in primary care patients with uninvestigated dyspepsia: the Canadian Adult Dyspepsia Empiric Treatment – Prompt Endoscopy (CADET-PE) study. Aliment Pharmacol Ther.

[6] Armstrong D, Marshall JK, Chiba N (2005). Canadian Consensus Conference on the management of gastroesophageal reflux disease in adults – update 2004. Can J Gastroenterol.

[7] DeVault KR, Castell DO (2005). Updated guidelines for the diagnosis and treatment of gastroesophageal reflux disease. Am J Gastroenterol.

[8] Dent J, Armstrong D, Delaney B (2004). Symptom evaluation in reflux disease: workshop background, processes, terminology, recommendations, and discussion outputs. Gut.

[9] Fock KM, Talley N, Hunt R (2004). Report of the Asia-Pacific consensus on the management of gastroesophageal reflux disease. J Gastroenterol Hepatol.

[10] Vakil N, Veldhuyzen van Zanten S, Kahrilas P (2006). The Montreal definition and classification of gastroesophageal reflux disease: a global evidence-based consensus. Am J Gastroenterol.

[11] Veldhuyzen van Zanten S, Armstrong D, Barkun A (2007). Symptom overlap in patients with upper gastrointestinal complaints in the Canadian confirmatory acid suppression test (CAST) study: further psychometric validation of the reflux disease questionnaire. Aliment Pharmacol Ther.

[12] Fass R, Achem SR, Harding S (2004). Review article: supra-oesophageal manifestations of gastro-oesophageal reflux disease and the role of night-time gastro-oesophageal reflux. Aliment Pharmacol Ther.

[13] Wiklund I (2004). Review of the quality of life and burden of illness in gastroesophageal reflux disease. Dig Dis.

[14] Patrick DL, Deyo RA (1989). Generic and disease-specific measures in assessing health status and quality of life. Med Care.

[15] Ware JE, Sherbourne CD (1992). The MOS 36-item short-form health survey (SF-36). I. Conceptual framework and item selection. Med Care.

[16] Dupuy HJ, Wenger NK, Mattson ME, Furberg CF (1984). The psychological general well being index. Assessment of Quality of Life in Clinical Trails of Cardiovascular Therapies.

[17] EuroQol Group (1990). EuroQol: a new facility for the measurement of health-related quality of life. Health Policy.

[18] Wiklund IK, Junghard O, Grace E (1998). Quality of life in reflux and dyspepsia patients. Psychometric documentation of a new disease-specific questionnaire (QOLRAD). Eur J Surg Suppl.

[19] Svedlund J, Sjodin I, Dotevall G (1988). GSRS – a clinical rating scale for gastrointestinal symptoms in patients with irritable bowel syndrome and peptic ulcer disease. Dig Dis Sci.

[20] Shaw MJ, Talley NJ, Beebe TJ (2001). Initial validation of a diagnostic questionnaire for gastroesophageal reflux disease. Am J Gastroenterol.

[21] Jones R, Coyne K, Wiklund I (2007). The Gastro-oesophageal Reflux Disease Impact Scale: a patient management tool for primary care. Aliment Pharmacol Ther.

[22] Revicki DA, Wood M, Wiklund I (1998). Reliability and validity of the gastrointestinal symptom rating scale in patients with gastroesophageal reflux disease. Qual Life Res.

[23] Kulig M, Leodolter A, Vieth M (2003). Quality of life in relation to symptoms in patients with gastro-oesophageal reflux disease – an analysis based on the ProGERD initiative. Aliment Pharmacol Ther.

[24] Kaplan-Machlis B, Spiegler GE, Revicki DA (1999). Health-related quality of life in primary care patients with gastroesophageal reflux disease. Ann Pharmacother.

[25] Liker H, Hungin P, Wiklund I (2005). Managing gastroesophageal reflux disease in primary care: the patient perspective. J Am Board Fam Pract.

[26] Johnson DA (2005). Gastroesophageal reflux disease and sleep disorders: a wake-up call for physicians and their patients. Rev Gastroenterol Disord.

[27] National Heartburn Alliance (2000). National Heartburn Alliance Survey 2000 Results: A Community Perspective.

[28] Shaker R, Castell DO, Schoenfeld PS (2003). Nighttime heartburn is an under-appreciated clinical problem that impacts sleep and daytime function: the results of a Gallup survey conducted on behalf of the American Gastroenterological Association. Am J Gastroenterol.

[29] Farup C, Kleinman L, Sloan S (2001). The impact of nocturnal symptoms associated with gastroesophageal reflux disease on health-related quality of life. Arch Intern Med.

[30] Wiklund I, Carlsson J, Vakil N (2006). Gastroesophageal reflux symptoms and well-being in a random sample of the general population of a Swedish community. Am J Gastroenterol.

[31] Junghard O, Carlsson R, Lind T (2003). Sufficient control of heartburn in endoscopy-negative gastro-oesophageal reflux disease trials. Scand J Gastroenterol.

[32] Hunt RH (1995). The relationship between the control of pH and healing and symptom relief in gastro-oesophageal reflux disease. Aliment Pharmacol Ther.

[33] Revicki DA, Sorensen S, Maton PN (1998). Health-related quality of life outcomes of omeprazole versus ranitidine in poorly responsive symptomatic gastroesophageal reflux disease. Dig Dis.

[34] Havelund T, Lind T, Wiklund I (1999). Quality of life in patients with heartburn but without esophagitis: effects of treatment with omeprazole. Am J Gastroenterol.

[35] Johanson JF, Siddique R, Damiano AM (2002). Rabeprazole improves health-related quality of life in patients with erosive gastroesophageal reflux disease. Dig Dis Sci.

[36] Damiano A, Siddique R, Xu X (2003). Reductions in symptom distress reported by patients with moderately severe, nonerosive gastroesophageal reflux disease treated with rabeprazole. Dig Dis Sci.

[37] Pare P, Armstrong D, Pericak D (2003). Pantoprazole rapidly improves health-related quality of life in patients with heartburn: a prospective, randomized, double blind comparative study with nizatidine. J Clin Gastroenterol.

[38] Revicki DA, Zodet MW, Joshua-Gotlib S (2003). Health-related quality of life improves with treatment-related GERD symptom resolution after adjusting for baseline severity. Health Qual Life Outcomes.

[39] Johnson DA, Orr WC, Crawley JA (2005). Effect of esomeprazole on nighttime heartburn and sleep quality in patients with GERD: a randomized, placebo-controlled trial. Am J Gastroenterol.

[40] de Souza Cury M, Ferrari AP, Ciconelli R (2006). Evaluation of health-related quality of life in gastroesophageal reflux disease patients before and after treatment with pantoprazole. Dis Esophagus.

[41] Hansen ÅN, Bergheim R, Fagertun H (2006). Long-term management of patients with symptoms of gastro-oesophageal reflux disease – a Norwegian randomised prospective study comparing the effects of esomeprazole and ranitidine treatment strategies on health-related quality of life in a general practitioners setting. Int J Clin Pract.

[42] Ponce J, Arguello L, Bastida G (2004). On-demand therapy with rabeprazole in nonerosive and erosive gastroesophageal reflux disease in clinical practice: effectiveness, health-related quality of life, and patient satisfaction. Dig Dis Sci.

[43] Fallone CA, Guyatt GH, Armstrong D (2004). Do physicians correctly assess patient symptom severity in gastro-oesophageal reflux disease. Aliment Pharmacol Ther.

[44] McColl E, Junghard O, Wiklund I (2005). Assessing symptoms in gastroesophageal reflux disease: how well do clinicians’ assessments agree with those of their patients. Am J Gastroenterol.

[45] Carlsson R, Dent J, Bolling-Sternevald E (1998). The usefulness of a structured questionnaire in the assessment of symptomatic gastroesophageal reflux disease. Scand J Gastroenterol.

[46] Crawley JA, Schmitt CM (2000). How satisfied are chronic heartburn sufferers with their prescription medications? Results of the Patient Unmet Needs Survey. J Clin Outcomes Manag.

[47] Degl’ Innocenti A, Guyatt GH, Wiklund I (2005). The influence of demographic factors and health-related quality of life on treatment satisfaction in patients with gastroesophageal reflux disease treated with esomeprazole. Health Qual Life Outcomes.

[48] Shikiar R, Flood E, Siddique R (2005). Development and validation of the gastroesophageal reflux disease treatment satisfaction questionnaire. Dig Dis Sci.

[49] Armstrong D, Veldhuyzen van Zanten SJ, Chung SA (2005). Validation of a short questionnaire in English and French for use in patients with persistent upper gastrointestinal symptoms despite proton pump inhibitor therapy: the PASS (Proton pump inhibitor Acid Suppression Symptom) test. Can J Gastroenterol.

